# Rare Virulences and Great Pathotype Diversity of a Central European *Blumeria hordei* Population

**DOI:** 10.3390/jof9111045

**Published:** 2023-10-25

**Authors:** Antonín Dreiseitl

**Affiliations:** Department of Integrated Plant Protection, Agrotest Fyto Ltd., 767 01 Kroměříž, Czech Republic; dreiseitl@vukrom.cz

**Keywords:** barley, *Blumeria graminis* f. sp. *hordei*, *Hordeum vulgare*, reverse octal notation, powdery mildew, resistance genes, virulence complexity, virulence frequency

## Abstract

Barley is an important crop grown on almost 49 Mha worldwide in 2021 and is particularly significant in Europe where powdery mildew is the most frequent disease on susceptible varieties. The most suitable way to protect crops is by exploiting genetic resistance. However, the causal agent *Blumeria hordei* is an extremely adaptable pathogen. The aims of this research were to increase our knowledge of the rapidly changing pathogen population and detect rare virulences. Random samples of the pathogen were obtained from the air by means of a mobile spore sampler. Spores were collected by driving across the Czech Republic in 2019, 2021 and 2023, and 299 isolates were analyzed on 121 host varieties. No infection occurred on 35 differentials, rare virulence was recorded on 31 varieties and a higher virulence frequency was found on 55 differentials. A core set of differentials along with four additional varieties distinguishes 295 pathotypes (Simple Index = 0.987) and the virulence complexity of isolates varied from 4 to 19 with an average of 10.39. The detection of new virulences, the increasing frequency of previously rare virulences and high pathotype diversity as well as high virulence complexity confirm that using nonspecific durable resistance is crucial for successfully breeding commercial varieties.

## 1. Introduction

Virulence of a pathotype (race) reflects the ability of a pathogen to infect a host variety carrying a gene of specific resistance against a respective disease. *Blumeria hordei* M. Liu & Hambl. (*Bh*) is an airborne biotrophic fungus causing powdery mildew (PM) predominantly on cultivated barley (*Hordeum vulgare* L.) and *H. murinum* L. [1]. Barley PM occurs everywhere barley is grown. In southeastern and southwestern China PM is a major airborne foliar disease of barley [2]. In the Qinghai-Tibet plateau barley is the most important cereal crop and in recent years yield has been seriously threatened by PM [3]. Among 41 barley diseases occurring in Australia, PM causes the second highest yield losses [4] and on varieties infected by PM, more lodging occurs with an associated effect on other traits including grain yield [5].

In Europe, and particularly in the Central and Northwest areas, PM is the most frequent disease of non-resistant barley varieties [6,7] and the crop is routinely protected against the disease. However, the optimum way to combat the pathogen is to grow resistant cultivars. Many effective resistances have been found in landraces [8,9,10] and especially in wild barley (*H. v.* subsp. *spontaneum*) [11,12,13] and have been used to breed commercial varieties [14,15,16].

*B. hordei* has the highest evolutionary potential of most pathogens of crop plants [17]. Hybridization within a PM species or *forma specialis* creates pathotype diversity and can lead to the emergence of new PM forms and an expanding host range [18,19]. Transposable elements play a key role in modifying the *Blumeria* genome and enable rapid adaptation of the pathogens towards overcoming plant immunity [20]. The host-specific resistance genes present in cultivated varieties result in the directional selection of corresponding virulences and thus leave traces in the pathogen population [21].

Monitoring PMs on different crops [22,23,24] allows comparison of local populations [2,3,25] or populations within [26] and between continents [27,28] including the detection of new virulences [29].

In Europe, 46% of the total world barley area was grown in 2021 [30] and, mainly in the central part of the continent, there is a high concentration of both winter and spring forms. Simultaneous cultivation of spring and winter barley carrying a diversity of different specific resistance genes, the presence of the host in the field through the year and the given favorable conditions for the pathogen development including unlimited migration create a unique foundation for model population studies.

The aims of the current research are first, to keep pace with the characterization of a rapidly adapting population, second, to understand the pathogen itself and third, to obtain essential isolates for resistance gene postulation [31]. Special attention was paid to detecting rare virulences to the corresponding resistances of the investigated varieties.

## 2. Materials and Methods

### 2.1. Host Plant Material

About 25 seeds of the susceptible Australian barley variety Stirling [32] were sown in a pot (80 mm diameter) filled with a gardening peat substrate and placed in a PM-proof greenhouse under natural daylight. Healthy primary leaves were excised when the second leaves were emerging, placed in 120 mm glass Petri dishes on water agar (0.8%) containing benzimidazole (40 mg L^−1^), a leaf senescence inhibitor, and inserted in the bottom of a spore sampler (BMF company, Kroměříž, Czech Republic).

One hundred and twenty-one barley differential varieties containing mostly different resistances against PM were sown as above. These were used to determine the corresponding virulences of individual isolates. Of these, 16 near-isogenic Pallas lines [33], 47 commercial varieties, 31 breeding lines and 27 other genotypes, mainly sources of resistance genes, were included. Seeds of single plant progenies [34] of all differentials had previously been produced.

### 2.2. Samples of the Pathogen Population

**Figure 1:**
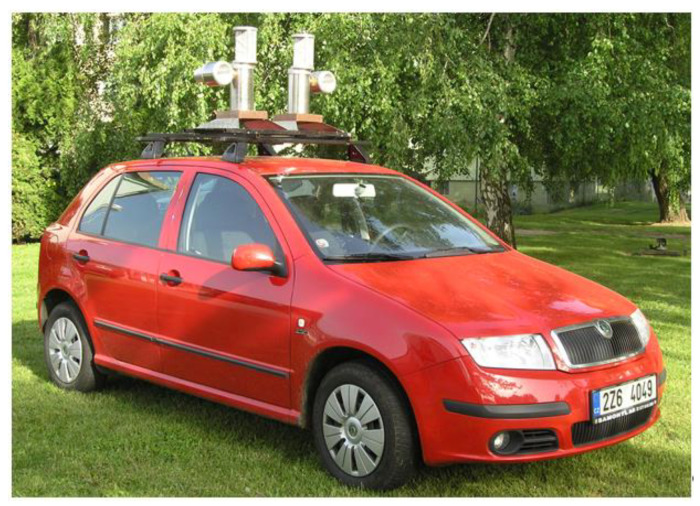
A car with a universal doubled jet spore sampler.

Random samples of the pathogen population originating from naturally infected spring and winter barley fields were obtained from the air by a universal doubled jet spore sampler [35] mounted on the roof of a car (Figure 1). Spores were collected by driving across the Czech Republic in three years (2019, 2021 and 2023) from mid-May to early June when tillering of spring barley had usually ended and winter barley was at the ear emergence stage.

While travelling, the collected spores settled on detached leaves of Stirling. Dishes were replaced for each 50–100 km section of the sampling route (Table 1) totalling annually to more than 1000 km. During sampling, dishes were kept in a car-refrigerator at about 8 °C. After sampling, exposed leaves were transferred to glass Petri dishes of 150 mm diameter with fresh agar. Dishes with detached leaves of the susceptible variety and settled spores were incubated for 11 to 13 days at 18.0 ± 1 °C under artificial light (cool-white fluorescent lamps providing 12 h light at 30 ± 5 μmol·m^−2^·s^−1^).

### 2.3. Testing Procedure

**Figure 2:**
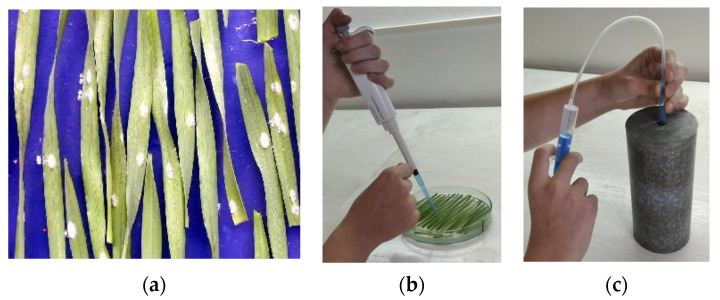
(**a**) Colonies of *Blumeria hordei* developed from single spores caught with a jet spore sampler; (**b**) Sucking a spore progeny conidia into an exchangeable tip of a varipipette; (**c**) Blowing conidia off the tip into a micro-settling tower.

Leaf segments of differentials 15 mm long were cut from the central part of healthy primary leaves of each variety. Testing of isolates on differentials was performed in two steps. First, two segments of standard near-isogenic lines were placed adjacently with the adaxial surface upward on agar media in 90 mm plastic Petri dishes. For inoculation, conidia from each single-spore colony (Figure 2a) were sucked into a replaceable tip of an AW 1000 varipipette (Figure 2b) and then blown off the tip into a micro-settling tower using a syringe connected to the tip with a plastic tube (Figure 2c). In this manner, spores were spread and settled on leaf segments of differentials in a Petri dish placed at the bottom of the tower. The inoculum density was usually about 5 conidia·mm^−2^. Dishes with inoculated differentials were incubated in the described conditions.

After evaluation of the first part of the differential set, a second inoculation was performed using conidiospores from nine-day-old colonies of isolates produced on susceptible varieties. For every isolate, a 150 mm glass Petri dish with a leaf segment of each variety of the second part of the differential set was placed at the bottom of the larger settling tower. Conidia of an isolate from an infected leaf segment were shaken onto a square piece of black paper to visually estimate the amount of inoculum deposited. This was then gently rolled to form a blowpipe and the conidia were blown into the settling tower over the Petri dish. The inoculum density was around 10 conidia·mm^−2^. The dishes with inoculated leaf segments were kept under the previously described incubation conditions.

### 2.4. Evaluation

Seven days after inoculation, infection response (IR = phenotype of a differential variety × isolate interaction) was scored on a scale of 0–4 [36], where 0 = no traces of the pathogen, and 4 = strong mycelial growth and sporulation. Isolates showing IRs 3, 3–4 and 4 were considered virulent. During phenotyping, special attention was paid to the boundary IRs 2–3 and 3 which pose the greatest risk of error in distinguishing between virulence and avirulence [37]. In doubtful cases regarding the virulence of the isolates, and in all cases when rare virulences were found (usually up to 3% of the frequency), re-inoculation was carried out. In accordance with the gene-for-gene concept [38] pathotypes were postulated [39] on the base of virulence/avirulence isolate phenotypes on the set of barley differentials ranked in the given order. Details of materials and methods including other demonstration images have been recently published [31].

### 2.5. Pathotype Classification

To classify isolates a set of differential varieties used in all three years was used. The numerical pathotype designation of isolates was based on their virulence/avirulence pattern on the set of 40 differentials divided into 13 triplets and the last variety on its own. Each of the digits indicates virulence to the three differentials of the respective triplet. If virulence to a corresponding variety was detected, the first differential is given the value 1 (2^0^), the second differential has the value 2 (2^1^), and the third differential is 4 (2^2^). Therefore, each digit can have a value from 0 (no virulence to any of the three differentials) up to 7 (1 + 2 + 4), denoting virulence to each of the three varieties [40]. The resulting number (reverse-octal) [41] defines the pathotype classification of isolates. The HaGiS program was used for the transcription of the infection response arrays (IRAs) into the notation [42].

## 3. Results

Over a three-year period, population samples of 299 isolates were studied on 121 host differentials. Of these, 37 differentials were used in two and 23 in only one year (Table 2). Hence, virulence frequency (VF) was determined on 95 differentials in the first year, on 92 in 2021 and on 93 host genotypes in the last year.

### 3.1. Virulence of Isolates and Virulence Frequency of the Population

In 2019 there were no virulent isolates recorded on 39 differentials (VF = 0%) and none on 31 differentials in 2021 and 2023 (=non-differentiating varieties).

In all three years, 61 differentials were used, but no virulence was detected on 14 of these (nos. 1–14). A rare virulence (VF 0.3–5.0%) was recorded on 18 host genotypes (nos. 15–32) and higher VF (8.7–98.7%) on 22 differentials (nos. 33–54). Seven differentials (SJ123063, SY412-329, Landi, NORD 14/1116, CH-666, Diabas and Kompolti 4; nos. 55–61) were omitted because they carried the same resistance as six other differentials, namely Zeppelin, which contains an identical resistance as SJ123063 and SY412-329, and Florian, Pop, P23, P04B and P15. Identical VFs were found on these. Sixty additional differentials were used in one or two years.

### 3.2. Pathotype Diversity

The virulence of isolates to resistance of differentials resulted in IRAs. For the numerical pathotype designation, the core set of 40 differentials (nos. 15–54) was arranged according to the ascending total VF. Based on their IRAs, 299 isolates were assigned to 291 pathotypes (Table S1) when each of six pathotypes (0001221, 0030571, 0005771, 0074671, 0076771 and 0147771) was represented by two and one pathotypes (2203271) by three isolates (note that in designating these pathotypes the first seven zeros were omitted here). Two pathotypes (0030571 and 0005771) found in 2019 were represented by two isolates (G-2, G-18 and R-4, R-28 respectively). In the same year, 34 additional differentials were used, and the first pair of isolates differed in virulence to A-222 and the second pair in virulence to Prosa. Two isolates (E-1 and R-5) found in 2023 belonged to pathotype 0147771 but they differed in virulence to the resistance of Mirko. Also, two other isolates (N-2 and O-1) out of three represented by pathotype 2203271 were found in 2023 and they differed in virulence to resistance of Gilberta. The third isolate (Y-1) was collected in 2021, and in 2021 and 2023 13 joint additional differentials were used (nos. 86–98). However, none of these could differentiate among these three isolates nor three other pairs of isolates found in 2021 and 2023 (C-12/2021, M-1/2023; I-1/2021, O-4/2023 and X-4/2021, F-1/2023). Hence, four pairs of isolates were differentiated using the named additional differentials and four were not. In summary, the given population sample of 299 isolates belonged to 295 pathotypes (Simple Index − SI = 0.987) (SI = the number of pathotypes/number of isolates) where almost every isolate belonged to a different pathotype.

### 3.3. Complexity of Virulences

The virulence complexity of isolates varied in a wide range continuously from 4 up to 19 with the exception of 5 (Table 3). The most numerous were isolates with a complexity of 8 up to 12 virulences and a frequency of 34–47 which included 204 (68.2%) isolates. The least common were isolates with extreme complexities 4 (2 isolates), 5 (0), 17 (2), 18 (1) and 19 (2). The average virulence complexity of isolates was 10.39.

### 3.4. Selection of Isolates for Resistance Gene Postulation

In total, 22 isolates were selected for future use in postulating resistance genes, five in 2019, eight in 2021 and nine in 2023 (Table S1). Isolates were chosen according to their rare virulences or suitable virulence combinations.

## 4. Discussion

This contribution completes more than six decades of monitoring of the *Bh* population conducted in our laboratory, and the research was always closely linked with the identification and use of new host-specific resistances [22,43], designation of new resistance genes [44,45] and resistance/gene postulation in varieties including those newly registered as commercial cultivars [46,47].

The last population study was performed on 50 differentials [21]; 47 of them were also used here, whereas two were excluded after identifying their resistance: NORD12/1122 which contained a gene of nonspecific resistance *mlo*, SJ048311 with a gene combination *Mlp*, *Mlat* and KM14/2010 was replaced with a sister line KM12/2010 for technical reasons.

Winter varieties Psaknon and Venezia were used as differentials for many years and the first virulences were found in 2011 (*VVe*) and 2012 (*Vp*) [29]. Nevertheless, corresponding VFs remained rare. Despite this, it was recognized that these virulences are associated [48,49]. In 2021 a greatly increasing number of both VFs was found and, therefore, three more varieties with *Mlp* (including Psaknon, which was not included in 2021) were added to the differential set in 2023 and a huge increase of both VFs was confirmed (*VVe* from 2.9% in 2019 to 26.2% in 2023 and *Vp* from 5.7% to 77.0%).

Saturn [47], a variety that was first registered in 2012 in the Czech Republic possesses *Mlp* and has occupied a negligible area. However, SU Ellen with the same gene and registered five years later [50] was the most widely grown winter barley variety in 2021 comprising around 10% of the crop area [51]. Therefore, it is likely that the directional selection and the migration of virulent pathotypes from surrounding countries, where varieties with *Mlp* had already begun to be cultivated, contributed to the dramatic increase in corresponding VF.

No variety carrying resistance of Venezia (*MlVe*) has been grown in the Czech Republic. Then the fast increase in frequency *VVe* could occur as a result of an increase in *Vp* due to hitch-hiking selection [52,53] and migration of the relevant virulence from neighboring countries.

Many differentials contain an unknown resistance, some of them possibly with the same major genes present in other varieties. Isolates selected here will serve as a useful tool for studying and identifying (postulation) their genes. At least 10 differentials should have SI-1 resistance, but the virulence of a few isolates differed in these as well as in some other varieties probably because of the presence/absence of some additional genes as was demonstrated on a set of varieties carrying resistance Lv [50]. Since 2012 six SI-1 differentials have been registered in the country but only Bente was grown on 1.7% in 2020 up to 3.9% of the spring barley area in 2023 [51]. VF to the resistance in Bente reached 6.6% and in Camilla 7.4% in 2023 and their resistance already has no importance in the field. New virulences were found on SJ123063, SY412-329 and KM12/2010.

Some other VFs differed non-significantly from a previous study [21], and it is difficult to explain these differences since many factors can play a role such as different evolutionary forces in the population, the epidemiological situation in different parts of the country, meteorological conditions during spore-trapping, etc. Some VFs showed a tendency to increase during the monitored period e.g., *Va1*, *Va3*, *Va7* or *VIM9*, whereas some decreased (*Va9*, *Vat* and *VRo*).

In previous tests, three varieties (Adam, Leenke and LG Nabuco) were resistant to all pathotypes, and their resistance gene(s) remained unknown (first two varieties) or unsure (LG Nabuco) [50]. A VF of 0% confirmed the complete effectiveness of their resistance and the occurrence of an IR0(2) enabled the presence of *mlo* to be deduced.

In 2023 Engledow India possessing a resistance gene designated *Mla24* [54] was included as a differential. Its IR and VF were identical to these parameters on P11 carrying *Mla13* [33]. It appears that both varieties have an identical resistance gene previously designated *Mla13* [55].

In 2016 and 2017, 226 isolates collected on an almost identical sampling route were analyzed and assigned to 224 pathotypes [21] (SI = 0.991). In this report, 299 isolates were studied in three years and 295 pathotypes were established (SI = 0.987). Thus, the present results confirmed an extremely high diversity of pathotypes that must be one of the highest among fungal plant pathogens. Two other characteristics are also high but further increasing, namely a wide spectrum of virulences and high virulence complexity of isolates. The causes of this situation have already been discussed [21], and apart from the high evolutionary potential of the pathogen, one must also consider the extensive cultivation of host crops, the continued use of a diverse set of resistances and long-term exploitation of specific resistances and suitable conditions for the pathogen development in the given area. The method of sampling spores (on average one analyzed isolate per >10 km of the sampling route) and the number of the selected host varieties with a known set of specific resistances in the differential set surely contributed to the discovery of the exceptional pathotype diversity.

Selected VFs found here can be compared with VFs in some non-European countries to show the role of directional selection in Central Europe (Table 4). High VF to the resistance gene *Ml(Ru2)* found in Chinese populations is also the result of directional selection because, in old Chinese barley varieties, this gene was found in 69 out of 147 tested accessions [56].

References of virulence to the nonspecific gene *mlo* are probably incorrect [2,3,59]. If this finding is correct, then the news should be widely disseminated to breeders and researchers so that remedial action can be taken. A similar announcement that avirulent isolates to the resistance gene *Mla8* had been discovered [59,60] has not been confirmed and there is only one known old pathotype (Race I) [61] available for specific research projects [62]. Unfortunately, most of the recent population studies [3,58,59] use only Pallas near-isogenic lines [33] carrying old “archival” resistance genes.

Our laboratory staff have studied *Bh* populations in all non-polar continents and the greatest differences in VFs and virulence complexity were found between Central European and Australian populations [27,57], and differences were confirmed with molecular characteristics of both numerous sets of isolates [28]. However, the results of another study of the Australian population were in many cases fundamentally different [60].

In population studies of plant pathogens, isolates should be properly designated reflecting their virulence combinations. Creating numerous systems has a long history and many of them have been developed, e.g., hexadecimal, based on 16 patterns designated with capital letters [63] that was subsequently used mainly in studies of cereal rusts, and an octal system based on a short, simple and logical mathematical row (see Materials and Methods, Section 2.5) [40], which was used preferably in PMs studies. A consensus of researchers agreed that modified octal notation (reverse octal notation) is the most appropriate for the given purpose [41] and it was recommended for general use [64]. Reverse octal notation has subsequently started to be employed for also designating resistance (IRAs) of host genotypes [12,13]. However, the hexadecimal system was recently proposed as “new” for characterizing oat PM pathotypes [65]. This contradicts the general recommendation and established methodology and is surely a retrograde step in plant pathological research.

Population studies should enable researchers to understand a pathogen and processes operating in its population. However, in many cases, the results are not comprehensible, and the conclusions of some papers are misleading, e.g., to designate resistance genes as “compromised” when corresponding VF is up to 50% [60], or to designate them as “highly resistant” even if the VF is about 10% [3]. Such statements can lead to false conclusions, overestimation of specific resistance and lead to the unsuccessful breeding of varieties resistant to the pathogen.

Specific resistance against barley PM can be an invaluable tool for pathologists, especially if based on genes expressing resistance as IR0 that maintains plants free from disease symptoms after inoculation with avirulent pathotypes. However, there are many examples of specific resistances being overcome in a short time (Table 5) and probably cannot maintain a sufficiently durable specific resistance even when more genes are combined (pyramided) in a variety. Therefore, until an effective way to prolong the durability of a specific resistance can be discovered, other possibilities should be explored [16]. These can be summarised as follows: (1) the wider exploitation of Mlo resistance especially outside Europe, (2) to determine whether Mlo resistance is a universal “weapon” against PM [66] and can be safely used in both forms of barley (winter and spring) in areas where they are intensively grown together, (3) to explore non-specific quantitative resistance genes, or (4) to exploit non-host resistance in updated meaning [67] derived from species naturally attacked by distantly related pathogens.

## Figures and Tables

**Table 1:** Sections of the sampling route for the collection of spores of *Blumeria hordei* from the air in the Czech Republic in 2019–2023 and the numbers of isolates studied.

Section of Sampling Route		Distance	Number of Isolates			
		km	2019	2021	2023	Sum
Brno–Břeclav	C	54	9	13	2	24
Brno–Kroměříž	A	68	9	9	17	35
Brno–Pardubice	S	70		17	39	56
Brno–Praha direction, motorway D1, 95. km	X	95	12	9	12	33
Brno–Znojmo	B	65	4	2	5	11
Kroměříž–Olomouc–Velký Újezd	R	92	37	7	5	49
Kroměříž–Otrokovice–Přerov	G	82	10		1	11
Olomouc–Šumperk	F	51	1	1	2	4
Ostrava–Velký Újezd	E	62	4	3	1	8
Praha–Brno direction, motorway D1, 95. km	I	90	1	2	3	6
Praha–Chomutov	U	75			12	12
Praha–Karlovy Vary	L	65	6	0	5	11
Praha–Pardubice	O	61	2	5	6	13
Praha–Plzeň	K	76	4	0		4
Praha–Turnov	N	68	1	2	2	5
Praha–Ústí nad Labem	M	70	5	2	10	17
Sum		1144	105	72	122	299

**Table 2:** Barley differential varieties, their *Ml* resistance genes and corresponding virulence frequency found in a Central European *Blumeria hordei* population.

No.	Differential Variety	Main	Virulence Frequency			
		*Ml* Gene(s)	2019	2021	2023	Total
1	Ab 1128	*ab*	0	0	0	0
2	Black Russian	*a2*	0	0	0	0
3	Bonita	*u*	0	0	0	0
4	Gopal	*a5*	0	0	0	0
5	Hb-81882/83	*hb1*	0	0	0	0
6	Hb-BC1-D5	*hb2*	0	0	0	0
7	LP 1506.1.96	*a3*, *aTu2*	0	0	0	0
8	Nigrate	*a30*	0	0	0	0
9	NORD 07017/69	*u*	0	0	0	0
10	NORD 18/2622	*u*	0	0	0	0
11	P13	*a23*	0	0	0	0
12	Sara	*a3*, *aTu2*	0	0	0	0
13	SK-4770-7	*g*, *u*	0	0	0	0
14	Spilka	*u*	0	0	0	0
15	Zeppelin selection	*SI-1*	0	1.4	0	0.3
16	Burštyn selection	*g*, *u*	1.0	1.4	0	0.7
17	GK Metal	*Ln*, *g*, *h*	1.0	1.4	0	0.7
18	HMK-8 selection	*g*, *u*	1.9	0	0.8	1.0
19	Klarinette	*SI-1*	1.9	1.4	0	1.0
20	KM-12/2010	*u*	0	1.4	1.6	1.0
21	SZD 3894	*u*	1.0	2.8	0	1.0
22	Florian	*Ln*	1.0	4.2	0	1.3
23	Pop	*SI-1*	0	2.8	1.6	1.3
24	Dubai	*u*	2.9	2.8	1.6	2.3
25	SBCC097	*Sb*	3.8	4.2	0	2.3
26	NORD 12101/116	*u*	1.9	.4.2	2.5	2.7
27	Remark	*SI-1*	0	2.8	4.9	2.7
28	SI-1	*SI-1*	0	4.2	5.7	3.3
29	Bente	*SI-1*	1.9	4.2	6.6	4.3
30	SU Celly	*u*	1.0	6.9	5.7	4.3
31	Camilla selection	*SI-1*	1.9	4.2	7.4	4.7
32	SU Laubella	*u*	1.0	6.9	7.4	5.0
33	P08B	*a9*	11.4	6.9	7.4	8.7
34	P20	*at*	17.4	11.1	5.7	11.0
35	KM-1867	*u*	6.7	16.7	15.6	12.7
36	Venezia selection	*Ve*	2.9	16.7	26.2	15.7
37	Laverda	*aLv*	13.3	15.3	21.3	17.1
38	P02	*a3*	17.1	19.4	16.4	17.4
39	Signal	*aN81*	23.8	19.4	22.1	22.1
40	P11	*a13*	29.5	25.0	27.9	27.8
41	P23	*La*	21.0	34.7	30.3	28.1
42	P17	*k1*	41.9	44.4	44.3	43.5
43	P09	*a10*	40.0	54.2	54.1	47.5
44	P19	*p1*	5.7	58.3	77.0	47.5
45	P12	*a22*	47.6	45.8	50.0	48.2
46	Alinghi	*IM9*	41.0	47.2	55.7	48.5
47	Annabell	*St*	49.5	63.9	40.2	49.2
48	Kangoo	*Ro*	69.5	61.1	43.4	56.9
49	P01	*a1*	47.6	56.9	65.6	57.2
50	P21	*g*	81.9	86.1	71.3	78.6
51	P04B	*a7*	80.0	83.3	88.5	84.3
52	P15	*Ru2*	73.3	97.2	91.8	86.6
53	P10	*a12*	92.4	90.3	91.8	91.6
54	P03	*a6*	96.2	100.0	100.0	98.7
55	SJ123063	*SI-1*	0	1.4	0	0.3
56	SY412-329	*SI-1*	0	1.4	0	0.3
57	Landi	*Ln*, *h*	1.0	4.2	0	1.3
58	NORD 14/1116	*u*	0	2.8	1.6	1.3
59	CH-666	*La*	21.0	34.7	30.3	27.8
60	Diabas	*a7*	80.0	83.3	88.5	84.3
61	Kompolti 4	*Ru2*	73.3	97.2	91.8	86.6
62	B-141/99	*a17*	0	0		0
63	C-213/01	*a26*	0	0		0
64	Hs HSY-78 × Aramir	*j*	0	0		0
65	Hs RS 110-4 × Sonja	*a29*	0	0		0
66	Hs RS 137-28 × Elgina	*f1*	0	0		0
67	Hs RS 142-29 × Dura	*a32*	0	0		0
68	Hs RS 145-39 × Kiebitz B	*a20*	0	0		0
69	Hs RS 170-10 × Piccolo A	*a25*	0	0		0
70	Hs RS 42-8 × Oriol A	*t*	0	0		0
71	Hs Diamant × 1B-86B	*a19*	1.0	0		0.6
72	Hs RS 170-47 × Kiebitz B	*a17*	0	1.4		0.6
73	E-388/01	*u*	0	2.8		1.1
74	Prosa	*u*	10.5	23.6		15.8
75	KM-1998	*u*	7.6	31.9		17.5
76	A222	*a11*	7.6		4.9	6.2
77	Meltan selection	*a13*, *Hu2*	16.2		10.7	13.2
78	Pribina	*a13*, *Hu2*	15.2		11.5	13.2
79	Souleyka	*aLv*	19.0		34.4	27.2
80	STRG 576/15	*aLv*	21.0		33.6	27.8
81	Traminer	*St*, *IM9*	37.1		38.5	37.9
82	Psaknon	*p1*	4.8		68.8	39.2
83	Klimek	*p1*	5.7		72.1	41.4
84	Amazone	*St*	45.7		38.5	41.8
85	Pionier	*Ro*	72.4		38.5	54.2
86	Adam	*mlo*		0	0	0
87	HOR2573	*La-H*		0	0	0
88	Kairyobozu-mugi	*kb*		0	0	0
89	LG Nabuco	*mlo*		0	0	0
90	SZD 5014A	*u*		0	0	0
91	Focus	*SI-1*		2.8	0	1.0
92	NOS 111.336-62	*u*		2.8	0	1.0
93	SG-S717-18	*u*		4.2	2.5	3.1
94	Padura	*u*		6.9	5.7	6.2
95	Torpedo	*u*		6.9	5.7	6.2
96	Maridol	*aN81*, *La*		5.6	7.4	6.7
97	Hulda	*a7*, *k1*		13.9	10.7	11.9
98	KM-2161	*u*		20.8	11.5	15.0
99	AC 07/624/34	*a3*, *aTu2*	0			
100	D-535/98	*a17*	0			
101	Hb-BC1-D27	*hb2*	0			
102	Hs Diamant × 1B-20	*a26*	0			
103	KM-1244	*a3*, *aTu2*	0			
104	KM-14/2010	*u*	0			
105	HE 1051	*u*	8.6			
106	Oowajao	*u*	11.4			
107	Black Heart	*u*	23.8			
108	Ricus	*u*	59.0			
109	Leenke	*mlo*		0		
110	Newton	*u*		4.2		
111	SZD 5111	*u*		8.3		
112	HM-407 selection	*u*		33.3		
113	SU Lauvira	*u*			0.8	
114	Nakaizumi-zairai	*k2*			4.1	
115	SC 21529 PH	*u*			5.7	
116	Chinerme	*p1*			14.8	
117	Engledow India	*a24*			27.9	
118	KM-2168	*u*			35.2	
119	Gilberta	*u*			62.3	
120	Mirko	*u*			79.5	
121	Tadmor	*aLo*			96.7	
	No. Differential Varieties		95	92	93	

**Table 3:** Virulence complexity and number of isolates found in a Central European population of *Blumeria hordei* in 2019–2023.

Virulence	No.	Sum of	Virulence	No.	Sum of
Complexity	Isolates	Isolate	Complexity	Isolates	Isolate
of Isolates		Virulences	of Isolates		Virulences
4	2	8	13	26	338
6	8	48	14	15	210
7	28	196	15	5	75
8	34	272	16	6	96
9	44	396	17	2	34
10	44	440	18	1	18
11	47	517	19	2	38
12	35	420	Sum	299	3106

**Table 4:** Selected virulence frequencies in some *Blumeria hordei* populations.

Differential	Main	Central	Australia ^2^	Kazakhstan ^3^	China	China	Turkey	Turkey
Variety	*Ml* Gene	Europe ^1^			South ^4^	Tibet ^5^	Adana ^6^	Hatay ^6^
P01	*a1*	57.2	0	0	0	0	0	0
P03	*a6*	98.7	0	0	10.6	0	36.7	27.9
P04B	*a7*	84.3	0	0	3.7	0	12.7	4.4
P10	*a12*	91.6	0	1.9	34.6	0.7	31.0	33.8
P11	*a13*	27.8	0	0	0.5	23.2	2.8	4.4
P21	*g*	78.6	79.5	0.9	21.8	20.8	19.7	8.8
P15	*Ru2*	86.6			95.7	76.9	25.3	16.2
P22	*mlo*	0	0	0	4.8	8.6	4.2	14.7
Pallas	*a8*	100	100	100	100	100	94.3	91.1

^1^ [this contribution], ^2^ [57], ^3^ [58], ^4^ [2], ^5^ [3], ^6^ [59].

**Table 5:** Breakdown of powdery mildew resistances of barley varieties carrying specific resistance gene(s) in Czech registration trials due to adaptation of the pathogen (*Blumeria hordei*) [68].

Variety	Year of	*Ml* Resistance	Average Resistance in Field Trials			
	Registration	Gene(s)	Highest		Lowest	
Ametyst	1972	*a6*	1971	7.20	1977	4.33
Trumpf	1976	*a7*, *aTr3*, *Ab*	1975	8.86	1979	5.44
Spartan	1977	*a9*	1976	8.60	1983	3.38
Zefir	1981	*a12*	1978	7.00	1986	2.50
Koral	1978	*a13*, *g*	1982	9.00	1986	5.50

## Data Availability

All relevant data are presented in the article and Table S1.

## Supplementary Materials

The following supporting information can be downloaded at: https://www.mdpi.com/article/10.3390/jof9111045/s1. Table S1: Year of sampling a Central European *Blumeria hordei* population, isolate and pathotype designation and their virulence complexity.

## References

[1] Liu M., Braun U., Takamatsu S., Hambleton S., Shoukouhi P., Bisson K.R., Hubbard K. (2021). Taxonomic revision of Blumeria based on multi-gene DNA sequences, host preferences and morphology. Mycoscience.

[2] Wang Y., Zhang G., Wang F., Lang X., Zhao X., Zhu J., Hu C., Hu J., Zhang Y., Yao X. (2023). Virulence variability and genetic diversity in *Blumeria graminis* f. sp. *hordei* in Southeastern and Southwestern China. Plant Dis..

[3] Wang Y.J., Zhuoma Q., Xu Z., Peng Y.L., Wang M. (2023). Virulence and genetic types of *Blumeria graminis* f. sp. *hordei* in Tibet and surrounding areas. J. Fungi.

[4] Murray G.M., Brennan J.P. (2010). Estimating disease losses to the Australian barley industry. Aust. Plant Pathol..

[5] Marzani Q.A., Amin M.M., Fateh S.A. (2023). Evaluation the effects of powdery mildew caused by *Blumeria graminis* f. sp. *hordei* and cultivar on the barley lodging. Eur. J. Plant Pathol..

[6] Jensen H.P., Christensen E., Jørgensen J.H. (1992). Powdery mildew resistance genes in 127 northwest European spring barley varieties. Plant Breed..

[7] Dreiseitl A. (2011). Diferences in powdery mildew epidemics in spring and winter barley based on 30-year variety trials. Ann. Appl. Biol..

[8] Jørgensen J.H., Jensen H.P. (1997). Powdery mildew resistance in barley landrace material 1. Screening for resistance. Euphytica.

[9] Czembor J.H., Czembor H.J. (2000). Powdery mildew resistance in selections from Moroccan barley landraces. Phytoparasitica.

[10] Czembor J.H., Czembor H.J. (2002). Selections from barley landrace collected in Libya as new sources of efective resistance to powdery mildew (*Blumeria graminis* f. sp. *hordei*). Rostl. Vyrob..

[11] Fischbeck G., Schwarzbach E., Sobel Z., Wahl I. (1976). Mildew resistance in Israeli populations of 2-rowed wild barley (*Hordeum spontaneum*). Z. Pflanz..

[12] Dreiseitl A., Dinoor A. (2004). Phenotypic diversity of barley powdery mildew resistance sources. Genet. Resour. Crop Evol..

[13] Dreiseitl A. (2017). Heterogeneity of powdery mildew resistance revealed in accessions of the ICARDA wild barley collection. Front. Plant Sci..

[14] Brown J.K.M., Jørgensen J.H., Jørgensen J.H. (1991). A catalogue of mildew resistance genes in European barley varieties. Integrated Control of Cereal Mildews: Virulence and Their Change, Proceedings of the Second European Workshop on Integrated Control of Cereal Mildews, Risø National Laboratory, Roskilde, Denmark, 23–25 January 1990.

[15] Jørgensen J.H. (1994). Genetics of powdery mildew resistance in barley. Crit. Rev. Plant Sci..

[16] Dreiseitl A. (2020). Specific resistance of barley to powdery mildew, its use and beyond. A concise critical review. Genes.

[17] McDonald B.A., Linde C. (2002). Pathogen population genetics, evolutionary potential, and durable resistance. Annu. Rev. Phytopathol..

[18] Praz C.R., Menardo F., Robinson M.D., Mueller M.C., Wicker T., Bourras S., Keller B. (2018). Non-parent of origin expression of numerous effector genes indicates a role of gene regulation in host adaption of the hybrid triticale powdery mildew pathogen. Front. Plant Sci..

[19] Muller M.C., Kunz L., Graf J., Schudel S., Keller B. (2021). Host adaptation through hybridization: Genome analysis of triticale powdery mildew reveals unique combination of lineage-specific effectors. Molec. Plant-Microbe Interact..

[20] Kusch S., Qian J., Loos A., Kuemmel F., Spanu P.D., Panstruga R. (2023). Long-term and rapid evolution in powdery mildew fungi. Molec. Ecology.

[21] Dreiseitl A. (2019). Great pathotype diversity and reduced virulence complexity in a Central European population of *Blumeria graminis* f. sp. *hordei* in 2015–2017. Eur. J. Plant Pathol..

[22] Dreiseitl A. (2008). Virulence frequency to powdery mildew resistances in winter barley cultivars. Czech J. Genet. Plant Breed..

[23] Czembor H.J., Domeradzka O., Czembor J.H., Mankowski D.R. (2014). Virulence structure of the powdery mildew (*Blumeria graminis*) population occurring on triticale (x triticosecale) in Poland. J. Phytopathol..

[24] Lalosevic M., Jevtic R., Zupunski V., Masirevic S., Orbovic B. (2022). Virulence structure of the wheat powdery mildew population in Serbia. Agronomy.

[25] Cieplak M., Nucia A., Ociepa T., Okon S. (2022). Virulence structure and genetic diversity of *Blumeria graminis* f. sp. *avenae* from different regions of Europe. Plants.

[26] Hovmøller M.S., Caffier V., Jalli M., Anderson O., Besenhofer G., Czembor J.H., Dreiseitl A., Felsenstein F., Fleck A., Heinrics F. (2000). The European barley powdery mildew virulence survey and disease nursery 1993–1999. Agronomie.

[27] Dreiseitl A. (2014). Pathogenic divergence of Central European and Australian populations of *Blumeria graminis* f. sp. *hordei*. Ann. Appl. Biol..

[28] Komínková E., Dreiseitl A., Malečková E., Doležel J., Valárik M. (2016). Genetic diversity of *Blumeria graminis* f. sp. *hordei* in Central Europe and its comparison with Australian population. PLoS ONE.

[29] Dreiseitl A. (2015). Rare virulences of barley powdery mildew found in aerial populations in the Czech Republic from 2009 to 2014. Czech J. Genet. Plant Breed..

[30] FAOSTAT. https://www.fao.org/faostat/en/.

[31] Dreiseitl A. (2022). Postulation of specific powdery mildew resistance genes in cereals: A widely used method and its detailed description. Pathogens.

[32] Dreiseitl A., Platz G. (2012). Powdery mildew resistance genes in barley varieties grown in Australia. Crop Pasture Sci..

[33] Kølster P., Munk L., Stølen O., Løhde J. (1986). Near-isogenic barley lines with genes for resistance to powdery mildew. Crop Sci..

[34] Dreiseitl A., Nesvadba Z. (2021). Powdery mildew resistance genes in single-plant progenies derived from accessions of a winter barley core collection. Plants.

[35] Schwarzbach E. (1979). A high throughput jet trap for collecting mildew spores on living leaves. Phytopathol. Z..

[36] Torp J., Jensen H.P., Jørgensen J.H. (1978). Powdery Mildew Resistance Genes in 106 Northwest European Spring Barley Cultivars. Year-Book, 1978.

[37] Kosman E., Chen X., Dreiseitl A., McCallum B., Lebeda A., Ben-Yehuda P., Gultyaeva E., Manisterski J. (2019). Functional variation of plant-pathogen interactions: New concept and methods for virulence data analyses. Phytopathology.

[38] Flor H.H. (1971). Current status of the gene-for-gene concept. Annu. Rev. Phytopathol..

[39] McVey D.V., Roelfs A.P. (1975). Postulation of genes for stem rust resistance in the entries of the Fourth international winter wheat performance nursery. Crop Sci..

[40] Gilmour J. (1973). Octal notation for designating physiologic races of plant pathogens. Nature.

[41] Limpert E., Müller K. (1994). Designation of pathotypes of plant pathogens. J. Phytopathol..

[42] Herrmann A., Löwer C.F., Schachtel G.A. (1999). A new tool for entry and analysis of virulence data for plant pathogens. Plant Pathol..

[43] Brückner F. (1963). Powdery mildew (*Erysiphe graminis* DC.) on barley. III. Investigation of physiological races of *Erysiphe graminis* DC. Detected in Czechoslovakia in 1960–61. Rostl. Vyr..

[44] Brückner F. (1982). The finding of powdery mildew (*Erysiphe graminis* DC. var. *hordei* Marchal) race on barley: A race virulent to resistance genes *Mla9* and *Mla14*. Ochrana Rostl..

[45] Dreiseitl A. (2011). Resistance of ‘Roxana’ to powdery mildew and its presence in some European spring barley cultivars. Plant Breed..

[46] Brückner F. (1964). Powdery mildew (*Erysiphe graminis* DC.) on barley. V. The resistance of barley varieties to physiological races of *Erysiphe graminis* DC. detected in Czechoslovakia and the possibility to use it in breeding for resistance. Rostl. Vyr..

[47] Dreiseitl A. (2017). Genes for resistance to powdery mildew in European barley cultivars registered in the Czech Republic from 2011 to 2015. Plant Breed..

[48] Dreiseitl A. (2016). Emerging *Blumeria graminis* f. sp. *hordei* pathotypes reveal ‘Psaknon’ resistance in European barley varieties. J. Agric. Sci..

[49] Dreiseitl A. (2018). Resistance of barley variety ‘Venezia’ and its reflection in *Blumeria graminis* f. sp. *hordei* population. Euphytica.

[50] Dreiseitl A. (2022). Powdery mildew resistance genes in European barley cultivars registered in the Czech Republic from 2016 to 2020. Genes.

[51] Obilniny 2023. https://eagri.cz/public/web/file/724909/Obilniny_2023.pdf.

[52] Brown J.K.M. (1995). Recombination and selection in populations of plant pathogens. Plant Pathol..

[53] Huang R., Kranz J., Welz H.G. (1995). Virulence gene-frequency change in *Erysiphe graminis* f. sp. *hordei* due to selection by non-corresponding barley mildew resistance gene and hitchhiking. J. Phytopathol..

[54] Jahoor A., Stephan U., Fischbeck G. (1990). Study of powdery mildew resistance gene from ´Engledow India´. Barley Genet. Newslett..

[55] Giese H., Jensen H.P., Jørgensen J.H. (1980). Allelism of genes in the *Ml-a* locus. Barley Genet. Newslett..

[56] Dreiseitl A., Yang J. (2007). Powdery mildew resistance in a collection of Chinese barley varieties. Genet. Resour. Crop Evol..

[57] Dreiseitl A., Fowler R.A., Platz G.J. (2013). Pathogenicity of *Blumeria graminis* f. sp. *hordei* in Australia in 2010 and 2011. Australas. Plant Pathol..

[58] Rsaliyev A., Pahratdinova Z., Rsaliyev S. (2017). Characterizing the pathotype structure of barley powdery mildew and effectiveness of resistance genes to this pathogen in Kazakhstan. BMC Plant Biol..

[59] Zeybek A., Khan M.K., Pandey A., Gunel A., Erdogan O., Akkaya M.S. (2017). Genetic structure of powdery mildew disease pathogen *Blumeria graminis* f. sp. *hordei* in the barley fields of Cukurova in Turkey. Fresenius Environ. Bull..

[60] Tucker M.A., Jayasena K., Ellwood S.R., Oliver R.P. (2013). Pathotype variation of barley powdery mildew in Western Australia. Australas. Plant Pathol..

[61] Hiura U., Heta H. (1955). Studies on the disease resistance in barley. III. Further studies on the physiologic races of *Erysiphe graminis hordei* in Japan. Ber. Des Ohara Inst. Für Landwirtsch. Biol..

[62] Bettgenhaeuser J., Hernández-Pinzón I., Dawson A.M., Gardiner M., Green P., Taylor J., Smoker M., Ferguson J.N., Emmrich P., Hubbard A. (2021). The barley immune receptor *Mla* recognizes multiple pathogens and contributes to host range dynamics. Nat. Commun..

[63] Roelfs A., McVey D.V. (1972). Wheat stem rust races in Yaqui valley of Mexico during 1972. Plant Dis. Report..

[64] Limpert E., Clifford B., Dreiseitl A., Johnson R., Müller K., Roelfs A., Wellings C. (1994). Systems of designation of pathotypes of plant pathogens. J. Phytopathol..

[65] Okon S., Cieplak M., Kuzdralinski A., Ociepa T. (2021). New pathotype nomenclature for better characterisation the virulence and diversity of *Blumeria graminis* f. sp. *avenae* populations. Agronomy.

[66] Kusch S., Panstruga R. (2017). *mlo*-based resistance: An apparently universal “weapon” to defeat powdery mildew disease. Molec. Plant-Microbe Interact..

[67] Panstruga R., Moscou M. (2020). What is the molecular basis of nonhost resistance?. Molec. Plant-Microbe Interact..

[68] Dreiseitl A. (2003). Adaptation of *Blumeria graminis* f. sp. *hordei* to barley resistance genes in the Czech Republic in 1971–2000. Plant Soil Environ..

